# Caspase-9 mediates synaptic plasticity and memory deficits of Danish dementia knock-in mice: caspase-9 inhibition provides therapeutic protection

**DOI:** 10.1186/1750-1326-7-60

**Published:** 2012-12-10

**Authors:** Robert Tamayev, Nsikan Akpan, Ottavio Arancio, Carol M Troy, Luciano D’Adamio

**Affiliations:** 1 Department of Microbiology & Immunology, Albert Einstein College of Medicine, Bronx, NY, 10461, USA; 2 Departments of Pathology & Cell Biology, Columbia University, New York, NY, 10032, USA; 3 Departments of Neurology, Columbia University, New York, NY, 10032, USA; 4 Departments of Taub Institute for the Study of Alzheimer’s Disease and the Aging Brain, Columbia University, New York, NY, 10032, USA

## Abstract

**Background:**

Mutations in either *Aβ Precursor protein* (*APP*) or genes that regulate APP processing, such as *BRI2/ITM2B and PSEN1*/*PSEN2*, cause familial dementias. Although dementias due to *APP*/*PSEN1*/*PSEN2* mutations are classified as familial Alzheimer disease (FAD) and those due to mutations in *BRI2/ITM2B* as British and Danish dementias (FBD, FDD), data suggest that these diseases have a common pathogenesis involving toxic APP metabolites. It was previously shown that FAD mutations in *APP* and *PSENs* promote activation of caspases leading to the hypothesis that aberrant caspase activation could participate in AD pathogenesis.

**Results:**

Here, we tested whether a similar mechanism applies to the Danish *BRI2/ITM2B* mutation. We have generated a genetically congruous mouse model of FDD, called FDD_KI_, which presents memory and synaptic plasticity deficits. We found that caspase-9 is activated in hippocampal synaptic fractions of FDD_KI_ mice and inhibition of caspase-9 activity rescues both synaptic plasticity and memory deficits.

**Conclusion:**

These data directly implicate caspase-9 in the pathogenesis of Danish dementia and suggest that reducing caspase-9 activity is a valid therapeutic approach to treating human dementias.

## Background

The prevailing pathogenic model for dementias caused by mutations in *APP* and genes that regulate APP processing (*PSEN1, PSEN2* and *BRI2/ITM2b*) posits that amyloid peptides trigger dementia. In AD, the amyloid peptide is Aβ that derives from APP processing. β-cleavage of APP, which is inhibited by BRI2, yields amino-terminal-soluble APPβ (sAPPβ) and β-carboxyl-terminal fragments (β-CTF). Processing of β-CTF by the γ-secretase complex, of which PSEN1 and PSEN2 constitute the catalytic components, releases Aβ. In FDD and FBD the amyloidogenic peptides, called ADan and ABri respectively, are generated from the mutant BRI2 proteins*.*

To model FDD we generated FDD_KI_ mice that, like FDD patients [1], carry a wild type *Bri2/Itm2b* allele and a Danish mutated allele [2]. FDD_KI_ mice develop progressive synaptic and memory deficits due to loss of BRI2 protein [3]. Owing to the loss of BRI2, processing of APP is increased in FDD [4,5], and sAPPβ/β-CTF, but not Aβ, trigger memory and synaptic deficits of FDD_KI_ mice [4,6,7]. These observations are consistent with the recent findings that β-processing of APP, but not Aβ, triggers pathological modifications associated with AD in human neurons derived from both familial and sporadic AD cases [8] and that a mutation in *APP* that reduces the BACE1 cleavage of APP protect elderly individual from sporadic AD and normal memory loss associated with ageing [9]. These similarities suggest that FDD shares common pathogenic mechanisms with FAD, involving synaptic-toxic APP metabolites distinct from Aβ.

We and others have shown that FAD mutations in *APP* and *PSENs* could promote activation of caspases [10-14]. These observations suggested that activation of caspases could play a pathogenic role in AD. In the ensuing years, a vast literature has linked Aβ to caspase activation, especially caspase-3, but a functional link has not been proven [15]. However, other reports have indicated that APP metabolites derived either from sAPPβ or the intracellular portion of β-CTF, and distinct from Aβ, also can promote activation of caspases [16-19]. Most caspases are mainly involved in the orchestration of the controlled demise of a cell after an apoptotic signal. These caspases are divided into those that initiate the apoptotic cascade (caspase-2, -8, -9 and −10, “initiator” caspases) and those that that execute apoptosis (caspase-3, -6, and −7, “effector” caspases). Initiator caspases are usually activated by dimerization, while effector caspases are activated by cleavage by initiator caspases [20]. Several recent observations show that apoptotic caspases also regulate other pathways including synaptic plasticity [21]. Based on these observations we tested whether caspases take part in the pathogenesis of memory loss and synaptic plasticity deficits of FDD_KI_ mice.

## Results

### The caspase inhibitors Z-VAD-*fmk* and Z-LEHD-fmk, but not Z-DEVD-fmk, rescue the synaptic plasticity deficits of FDD_KI_ mice

**Figure 1:**
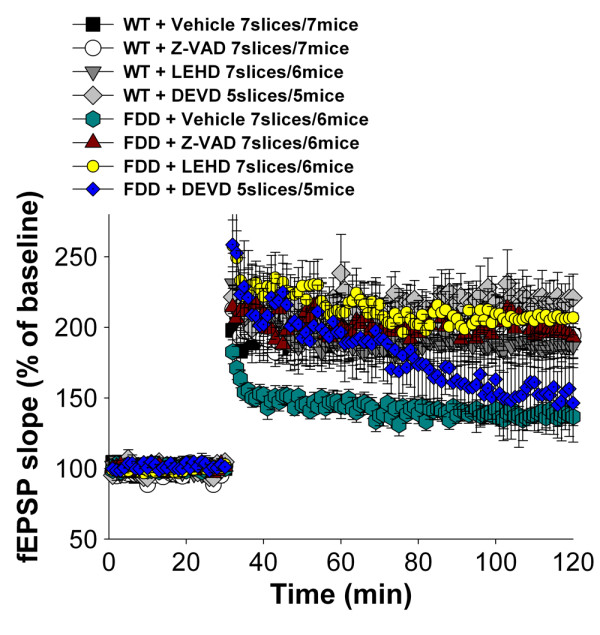
**Z-VAD-*fmk *and Z-LEHD-*fmk *rescue the synaptic deficits of FDD**_**KI **_**mice.** Vehicle-treated slices from FDD_KI_ mice exhibited a reduction in LTP compared to slices from vehicle-treated WT littermates [WT/vehicle *vs.* FDD/vehicle: F(1,12) = 27.008, P < 0.0001]. Perfusion with either 10 μM Z-VAD*-fmk* or 2 μM Z-LEHD*-fmk* reverses the LTP impairment of FDD_KI_ slices [WT/vehicle *vs.* FDD/Z-VAD-*fmk*: F(1,12) = 0.191, P = 0.671. FDD/vehicle *vs.* FDD/Z-VAD-*fmk*: F(1,12) = 14.300, P = 0.003. WT/vehicle *vs.* FDD/Z-LEHD-*fmk*: F(1,12) = 34.592, P < 0.0001. FDD/vehicle *vs.* FDD/Z-LEHD-*fmk*: F(1,12) = 34.592, P < 0.0001] but did not alter normal LTP responses in WT mice [WT/vehicle *vs.* WT/Z-VAD-*fmk*: F(1,12) = 0.032, P = 0.861. WT/vehicle *vs.* WT/ Z-LEHD-*fmk*: F(1,12) = 0.016, P = 0.900]. Differently, treating slices with 2 μM Z-DEVD*-fmk* did not overall rescue synaptic plasticity deficits of FDD_KI_ mice [WT/vehicle *vs.* FDD/Z-DEVD-*fmk*: F(1,12) = 0.191, P = 0.671. FDD/vehicle *vs.* FDD/Z-DEVD-*fmk*: F(1,10) = 4.272; P = 0.063], albeit it delayed the initiation of such deficits. In fact Z-DEVD-*fmk* rescued the LTP deficit during the initial 45 min of LTP [FDD/vehicle *vs.* FDD/Z-DEVD-*fmk*: F(1,10) = 8.93, P = 0.012], but not the deficit occurring during the last 45 min of LTP [FDD/vehicle *vs.* FDD/Z-DEVD-*fmk*: F(1,10) = 1.23, P = 0.29]. Of note, Z-DEVD*-fmk* did not alter LTP in WT mice [WT/vehicle *vs.* WT/Z-DEVD-*fmk*: F F(1,10) = 1.968, P = 0.191]. CA1-LTP was induced through a θ burst stimulation of the Shaffer collateral pathway.

In 1928 Ramon y Cajal predicted that weakening of synapses leads to dementia. Long-term potentiation (LTP) is a synaptic plasticity phenomenon that underlies the strengthening of synaptic functions during memory acquisition. Consistent with Ramon y Cajal’s prediction, LTP is defective in the hippocampal Schaffer collateral pathway of FDD_KI_ mice. However, basal synaptic transmission and paired-pulse facilitation are normal in FDD_KI_ mice, suggesting that no changes in Ca^2+^ mobilization or alterations in the probability of neurotransmitter release are driven by the Danish mutation [3]. To examine the role of caspases in synaptic plasticity, we analyzed the effect of the cell-permeable, irreversible pan-caspase inhibitor Z-VAD-*fmk* on LTP. Hippocampal slices were perfused either with Z-VAD-*fmk* (at 10 μM concentration) or vehicle for 60 min before inducing LTP. Z-VAD-*fmk* reversed the LTP deficit of Danish samples and did not alter LTP in wild-type mice (Figure 1).

Most caspases are expressed in the hippocampus. To start dissecting which caspase(s) play(s) a role in LTP deficits in FDD_KI_ mice, we analyzed the effect of Z-LEHD-*fmk* and Z-DEVD-*fmk*, which have partially overlapping inhibitory patterns of caspases inhibition. As shown in Figure 1, Z-LEHD-*fmk* behaved similarly to Z-VAD-*fmk* (i.e. it fully rescued the LTP deficit of FDD_KI_ mice, without imposing on normal synaptic plasticity). In contrast, LTP Z-DEVD-*fmk* delayed, but did not rescue, the insurgence of LTP deficits in FDD_KI_ mice (Figure 1). The evidence indicates that some, but perhaps not any, caspases are involved in the pathogenesis of LTP deficits of FDD_KI_ mice.

### The caspase inhibitor Z-LEHD-*fmk*, but not Z-DEVD-*fmk*, rescues the object recognition deficits of FDD_KI_ mice

**Figure 2:**
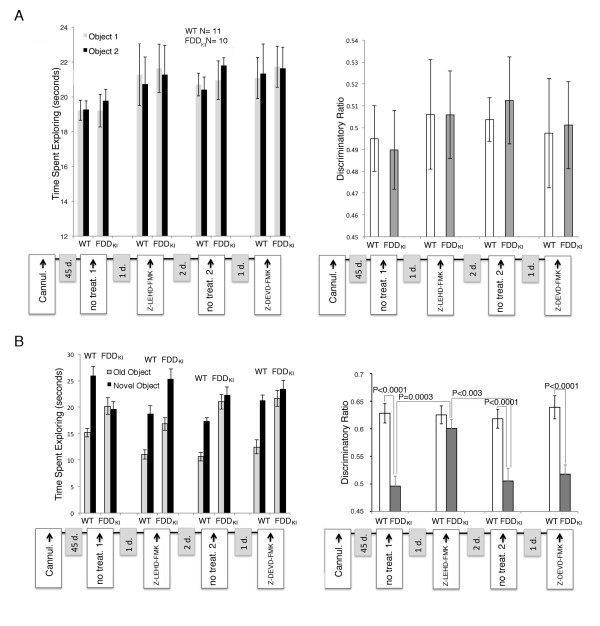
**The caspase inhibitor LEHD-*fmk *rescues the memory deficit of FDD**_**KI **_**mice.** Sequential NOR experiments on FDD_KI_ and WT mice. The first NOR was without treatment (no treat. 1); the second was performed after injecting in the lateral ventricle 1 μl of PBS-8%DMSO/800 μM Z-LEHD-*fmk* (Z-LEHD-*fmk*); the third NOR was without treatment (no treat. 2); the fourth was performed after injecting 1 μl of PBS-8%DMSO/800 μM Z-DEVD-*fmk* (Z-DEVD-*fmk*). The resting-days between each NOR are indicated in the X axis (1d., 2d. and 1d.). Training trials are reported in **A** as: Time-Spent-Exploring (left panel), discriminatory ratio (right panel), which is calculated as time-spent-exploring Object 1/time-spent-exploring Object 1+ time-spent-exploring Object 2. Data relative to the test trials are reported in **B**. The discriminatory ratio is calculated as time-spent-exploring the Novel Object/time-spent-exploring the Novel Object + time-spent-exploring Object 2. WT mice spent more time exploring the novel object showing normal object recognition, while FDD_KI_ mice present amnesia and do not distinguish the new object from the old one (B, no treat. 1). Notably, Z-LEHD-*fmk* rescues the deficit of FDD_KI_ mice while does not affect the NOR of WT mice (Z-LEHD-FMK in B). FDD_KI_ mice relapsed into amnesia (no treat. 2 in B), indicating that the therapeutic effect of Z-LEHD-*fmk* is short-lived. DEVD-*fmk* neither rescues the amnesia of FDD_KI_ mice nor it affects the NOR ability of WT mice (Z-DEVD-FMK in B). Objects were changed after each experiment. A paired two-sample t-test was used to test for significance between the discriminatory ratios between the groups.

We reasoned that if caspases have a causative role in dementia, then inhibiting caspase activity should in addition rescue memory deficits of FDD_KI_ mice. To test for this, we analyzed the effect of Z-LEHD-*fmk* and Z-DEVD-*fmk* on the memory deficits of FDD_KI_ mice in a longitudinal study. Memory was analyzed using novel object recognition (NOR), a non-aversive memory test that relies on the mouse’s natural exploratory behavior. The first NOR study showed that during training, FDD_KI_ and WT mice spent the same amount of time exploring two identical objects (Figure 2A, left panel), showing no discrimination between these two identical objects (Figure 2A, right panel). The following day, one of the two old objects was replaced with a new one to test the mouse’s memory. WT mice preferentially explored the novel object; conversely FDD_KI_ mice spent the same amount of time exploring the two objects as if they were both novel to them, showing that they had no memory of the objects from the previous day (Figure 2B, the left panel shows the time spent exploring each object, while the right panel show the discriminatory ratio). After this first test, the mice were rested for one day before re-testing. In this second experiment, the mice were injected in the lateral ventricle with Z-LEHD-*fmk* 1 h before the training/testing trials. Treated FDD_KI_ mice spent significantly more time exploring the novel object, just as treated controls did (Figure 2B). Following 2 days of rest, a new NOR test performed without treatments showed that FDD_KI_ mice had relapsed into amnesia (Figure 2B), demonstrating that the therapeutic effect of Z-LEHD-*fmk* is short-lived. One day later, mice were injected 1 h before the training/testing with Z-DEVD-*fmk*. Z-DEVD-*fmk* neither improved memory of FDD_KI_ mice nor altered performance of WT animals (Figure 2B). Thus, Z-LEHD-*fmk* (which fully corrected the synaptic deficit of FDD_KI_ mice) rescued, albeit temporarily, the memory deficit of FDD_KI_ mice. In contrast, Z-DEVD-*fmk* (which, as show in Figure 1, was inefficient in normalizing LTP in FDD_KI_ mice) did not. Although Z-LEHD-*fmk* and Z-DEVD-*fmk* are commonly referred to as specific caspase-9 and caspase3/7 inhibitors, respectively, these compounds show overlapping selectivity [22]. Therefore from these experiments it is difficult to pinpoint the caspase(s) involved in these pathogenic processes. However, altogether these data suggest that one or more caspases mediate synaptic/memory deficits of FDD_KI_ mice.

### The initiator caspase-9 is active in FDD_KI_ mice hippocampal synaptic fractions

**Figure 3:**
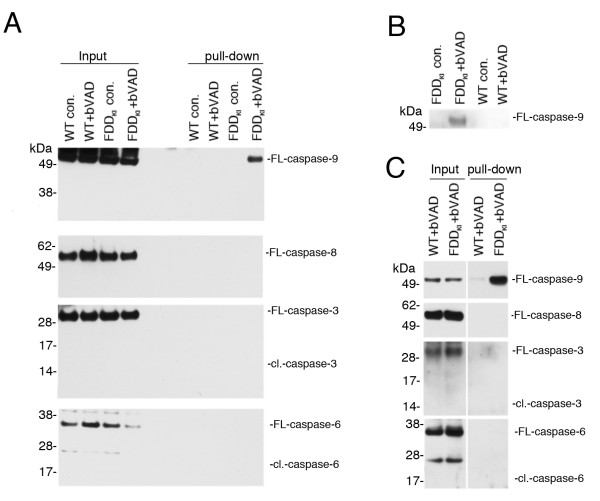
**High levels of active initiator caspase-9 in FDD**_**KI **_**mice.A**, Homogenates (input) were prepared from the bVAD injected (+bVAD) and contralateral non-injected (con.) hippocampal regions of WT and FDD_KI_ mice. Active caspases were isolated from homogenates with streptavidin-agarose-beads pull-down. Western blot analysis shows that the caspase inhibitor bVAD traps FL-caspase-9 only from the bVAD injected FDD_KI_ mouse hippocampus; FL-caspase-8, cl.caspase-3 and cl.-caspase-6 are not trapped. **B**, In a similar experiment, the streptavidin-agarose-beads pull-down experiment was performed from the P2 fractions. The P2 fractions represent crude synaptosomal fractions (see Material and Methods for details about these preparations). Again, active FL-caspase-9 is isolated from FDD_KI_ but not WT mice. **C**, Organotypic hippocampal cultures from either FDD_KI_ or WT mice were incubated for 3 hrs with 45 μM bVAD. After lysis, active caspases were isolated from homogenates. Again, caspase-9 was the only active caspase isolated. Albeit traces of active caspase-9 are found in the WT samples, the levels found in the FDD_KI_ hippocampus are greatly elevated. The blots shown in A, B and C are representative of duplicate experiments.

Based on the evidence that caspases are pathogenic in FDD_KI_ mice, we sought biochemical evidence of caspase activation and/or activity. Because FDD_KI_ mice have deficits in hippocampal-dependent memory and synaptic activity, which are associated with learning and memory, we tested whether we could detect signs of caspase activation in hippocampal synaptic preparations of 12 month-old mice. As discussed above, caspases are synthesized as zymogens (FL-caspase). Effector caspases are cleaved by initiator caspases (cl.-caspase) and this cleavage leads to activation of effector caspases. To allow unequivocal identification of active caspase we used an unbiased *in vivo* active caspase-trapping assay [23]. The caspase activity probe bVAD is the best way to determine whether caspases are active since bVAD binds irreversibly to all caspases that are active. In other words, if a caspase is active and its active site is available, bVAD will bind to it. Because bVAD is biotinylated, it can be isolated on streptavidin agarose along with any active caspase that is bound to it. This strategy has also the advantage of enriching for the apical active caspase rather than the downstream caspases in a pathway that involves a cascade of caspase activation. To determine which caspases are active, FDD_KI_ and WT mice were injected in one hippocampus with 100 nmol of bVAD. In these experiments, we utilized 6 (Figure 3A) or 5 (Figure 3B) month-old mice since the memory deficits of FDD_KI_ mice start at around 4–5 months of age [3]. Two hrs post treatment, the injected region and the contra-lateral non-injected area were dissected, and bVAD-caspase complexes were isolated on streptavidin-agarose beads and analyzed by Western blotting. bVAD captured greatly more FL-caspase-9, but not FL-caspase-8, from the hippocampus of the FDD_KI_ sample as compared to the WT littermate sample (Figure 3A). The binding was specific because streptavidin-agarose beads did not pull-down active FL-caspase-9 from homogenates prepared from the contra-lateral, non-injected sample. Cl.-caspase-3 and −6 were not trapped by bVAD (Figure 3A). The inability to isolate cl.-caspase-3 and cl.-caspase-6 may depend on the fact that bVAD inhibits caspase-9 activity, thereby inhibiting processing of effector caspases-3 and −6 by active caspase-9. This possibility is not very likely because in FDD_KI_ mice there is probably ongoing caspase activation and bVAD will bind to any active caspase present at the moment of bVAD administration. Alternatively, cl.-caspase-3 may not be available for bVAD-binding because it is complexed *in vivo* with endogenous inhibitor of apoptosis proteins (IAPs). Lastly, cl.-caspase-3 and cl.-caspase-6 may be captured by bVAD at very low levels that are below the detection power of our experimental system. This is indeed a possibility given the low level of material that can be harvested in this experimental setting and the evidence that cl-caspase-3 and cl-caspase-6 are not detectable in the input material either.

To determine whether active caspase-9 was present in synaptic fractions, we repeated the experiment and performed bVAD pull-downs from synaptosomal fractions. As shown in Figure 3B, active caspase-9 was also isolated from synaptosomal fractions of FDD_KI_ but not WT mice. Blotting for caspase-3, -6 and −8 showed once more absence of detectable active caspase-3, -6 or −8 in these synaptosomal preparations (data not shown). To formally exclude that the differences between WT and FDD_KI_ mice illustrated above did not depend on disparity of bVAD delivery *in vivo*, we prepared organotypic hippocampal cultures from 5 month-old WT and FDD_KI_ mice. BVAD trapped significantly more active caspase-9 from organotypic hippocampal culture of FDD_KI_ mice than WT littermates (Figure 3C). Once again, we could not detect active FL-caspase-8, cl.-caspase-3 and cl.-caspase-6 neither in WT nor in FDD_KI_ sample. Altogether these data indicate that caspase-9 is excessively activated in Danish dementia mice. Moreover, the data suggest that, if the Danish mutation triggers a cascade of caspase activation, caspase-9 is the apical caspase in such a cascade.

### Specific inhibition of caspase-9 with Pen1-XBIR3 provides therapeutic rescue of the object recognition deficit

**Figure 4:**
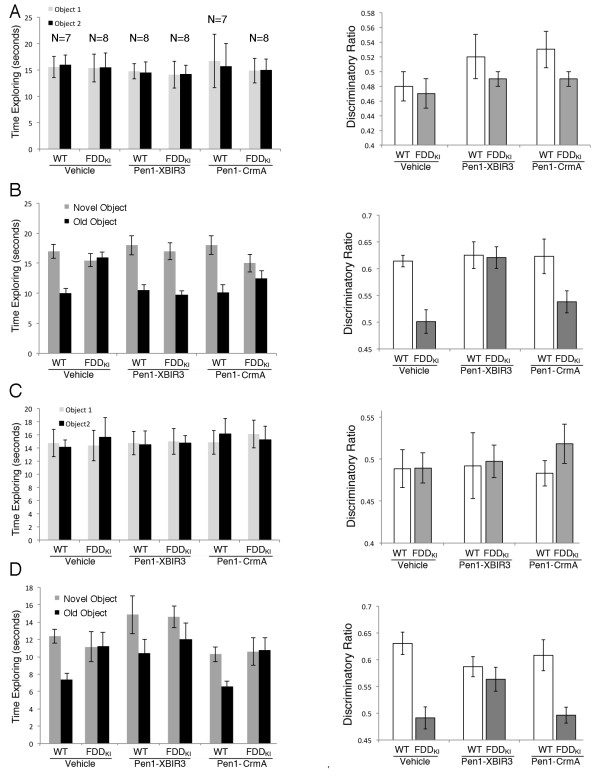
**Inhibition of caspase-9 with Pen1-XBIR3 rescues the memory deficits of FDD**_**KI **_**mice.** Twenty-five days after a cannula was implanted in the lateral ventricle, mice were injected in the lateral ventricle either with 2 μl of PBS/23 μM Pen1-XBIR3, 2 μl of PBS/16 μM Pen1-CrmA, or 2 μl of PBS alone (WT/PBS N = 7, WT/Pen1-XBIR3 N = 8, WT/Pen1-CrmA N = 7, FDD_KI_/PBS N = 8, FDD_KI_/Pen1-XBIR3 N = 8, FDD_KI_/Pen1-CrmA N = 8). Injections were performed 1 h prior to the training section and 1 h before testing. WT and FDD_KI_ mice spent the same amount of time exploring the two identical objects on day 1 (**A**). WT mice spent more time exploring the novel object 24 h later, while FDD_KI_ mice do not recognize the new object (WT/Vehicle *vs.* FDD_KI_/Vehicle P = 0.0007) . Pen1-XBIR3 rescues this memory deficit (FDD_KI_/Pen1-XBIR3 *vs.* WT/Vehicle P = 0.79; FDD_KI_/Pen1-XBIR3 *vs.* WT/Pen1-XBIR3 P = 0.89; FDD_KI_/Pen1-XBIR3 *vs.* WT/Pen1-CrmA P = 0.37; FDD_KI_/Pen1-XBIR3 *vs.* FDD_KI_ /Vehicle P = 0.0013; FDD_KI_/Pen1-XBIR3 *vs.* FDD_KI_/Pen1-CrmA P = 0.0027), while Pen1-CrmA does not (FDD_KI_/Pen1-CrmA *vs.* WT/Vehicle P = 0.0079; FDD_KI_/Pen 1-CrmA *vs.* WT/Pen1-XBIR3 P = 0.0038; FDD_KI_/Pen1-CrmA *vs.* WT/Pen1-CrmA P = 0.034; FDD_KI_/Pen1-CrmA *vs.* FDD_KI_ /Vehicle P = 0.24). (**B**). **C** and **D**, The NOR test was repeated 5 days later without further treatments. The therapeutic effect of Pen1-XBIR3 is still significant (WT/Vehicle *vs.* FDD_KI_/Vehicle P = 0.0003; FDD_KI_/Pen1-XBIR3 *vs.* WT/Vehicle P = 0.046; FDD_KI_/Pen1-XBIR3 *vs.* WT/Pen1-XBIR3 P = 0.44; FDD_KI_/Pen1-XBIR3 *vs.* WT/Pen1-CrmA P = 0.95; FDD_KI_/Pen1-XBIR3 *vs.* FDD_KI_ /Vehicle P = 0.03; FDD_KI_/Pen1-XBIR3 *vs.* FDD_KI_/Pen1-CrmA P = 0.028; FDD_KI_/Pen1-CrmA *vs.* WT/Vehicle P = 0.0002; FDD_KI_/Pen1-CrmA *vs.* WT/Pen1-XBIR3 P = 0.0025; FDD_KI_/Pen1-CrmA *vs.* WT/Pen1-CrmA P = 0.0038; FDD_KI_/Pen1-crmA *vs.* FDD_KI_/Vehicle P = 0.85). A paired two-sample t-test was used to test for significance between the discriminatory ratios between the groups.

The findings that reducing caspase activity with commercial peptide inhibitors rescues synaptic/memory deficits and that caspase-9 is active in FDD_KI_ mice, suggest that caspase-9 is involved in the pathogenesis of these deficits. To specifically determine the functional relevance of caspase-9 activity in memory loss pathogenesis, we specifically inhibited caspase-9. As a control, we also used a specific inhibitor of caspase-8 activity. Mammals express a family of cell death inhibiting proteins known as IAPs. IAPs contain BIR domains (Baculovirus Inhibitor of apoptosis protein Repeats), which perform specific functions. One member of this family, XIAP, is a potent specific inhibitor of active caspase-9, caspase-3, and caspase-7. The XIAP-BIR3 domain is a specific inhibitor of active caspase-9, and the XIAP-BIR2-linker domain inhibits active caspase-3 and caspase-7 [24]. Serpins are also caspases inhibitors and CrmA (a cowpox serpin) inhibits caspase-8 (as well as caspase-1, which is involved in inflammatory responses) but not other murine caspases [25]. To provide intracellular delivery, XIAP-BIR3 and CrmA were disulfide-linked to Penetratin1 (Pen1), a cell-penetrating peptide [23]. Upon entry into the cell the reducing environment of the cytoplasm reduces the disulfide linkage. This releases the peptide cargo and allows it to act at its target. We have previously shown that Pen1-XBIR3 inhibits caspase-9 dependent cell death using primary hippocampal neuron cultures, and that Pen1-XBIR3 delivery to the CNS blocks caspase-9 in an *in vivo* model of cerebral ischemia [23]. NOR experiments were used to assess the effect of Pen1-XBIR3 on memory. Six groups of mice (3 groups of FDD_KI_ mice and 3 groups of WT littermates) were injected in the lateral ventricle either with vehicle alone, Pen1-XBIR3 or Pen1-CrmA 1 hr before the training/testing trials. Pen1-XBIR3 treated FDD_KI_ mice spent significantly more time exploring the novel object showing reversal of the memory deficits (Figure 4A and B). On the contrary, Pen1-CrmA treated FDD_KI_ mice showed memory deficits comparable to those observed in vehicle-treated FDD_KI_ mice. Neither Pen1-XBIR3 nor Pen1-CrmA altered memory in WT animals. Following 5 days of rest, a new NOR test performed without treatments showed that the therapeutic effect of Pen1-XBIR3 persisted for at least 5 days post injection (Figure 4C and D). Our previous studies showed that one dose of Pen1-XBIR3 provided functional protection against ischemia for 3 weeks post-infarction [23]. Thus, Pen1-XBIR3 rescued the memory deficit of FDD_KI_ mice, while Pen1-CrmA did not. These data indicate that excessive activation of caspase-9 in FDD_KI_ mice is an essential step in the pathogenesis of memory loss.

## Discussion

We have tested whether caspases are involved in the pathogenesis of synaptic plasticity deficits and memory loss in FDD_KI_ mice and have used an unbiased approach to identify caspases that are critical for these pathological processes. Our data show that caspase-9 is a mediator of synaptic plasticity and memory deficits in FDD_KI_ mice. We have used active caspase trapping with bVAD, an irreversible pan-caspase inhibitor. This method provides a reliable measurement of caspase activity through biochemical pull-down of active caspases and has been shown to isolate active caspases-2, -3, -7 -8, or −9 from cell lines [26], in primary neuron cultures [27] and *in vivo* in the CNS [23]. With this method we show that FDD_KI_ mice have high levels of active caspase-9 in hippocampal synaptosomes. This is the first demonstration of a catalytically active initiator caspase in the hippocampus of animal models of familial dementia.

**Figure 5:**
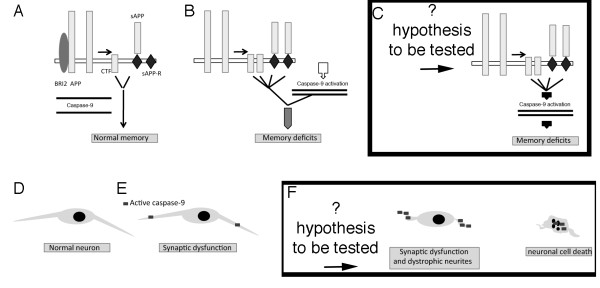
**Hypothetical model depicting the mechanisms by which caspase-9 can lead to alteration typical of neurodegenerative disorders: memory loss, dystrophic neurites and neuronal loss.A** and **B**, Previous work from our laboratory has shown that due to loss of BRI2 protein (loss of function model), APP processing is increased during synaptic transmission and memory acquisition in FDD leading to increased production of sAPPβ and β-CTF, leading to synaptic/memory deficits. We now show that caspase-9 is activated, via and unknown mechanisms, in FDD. This increased caspase-9 activation leads to synaptic/memory deficits via a yet to be defined mechanism. In **C**, we postulate a hypothetical pathway in which caspase-9 is activated by β-CTF and/or sAPPβ, perhaps via interaction with a membrane-bound receptor, sAPPβ-R, such as DR6 [19]. Whether sAPPα and/or α-CTF can also trigger this pathway remains to be determined. In this context, it is worth noting that BRI2 also inhibits α-secretase processing of APP [4,28,29]. Further studies will be needed to assess the role of the α-processing pathway of APP in dementia. **D** and **E**, Aberrant activation of caspase-9 in synaptosomes causes functional impairments leading to synaptic plasticity and memory acquisition deficits, with no noticeable anatomical changes. In F, we hypothesize that repetitive cycles of high caspase-9 activity can lead to dystrophy of neurites. Prolonged and sustained activation of caspase-9 increases the probability that in any given neuron caspase-9 activity may leak to the cell body and prompt the demise of the neuron.

Inhibiting caspases with commercial peptide inhibitors reversed synaptic plasticity deficits and memory loss in FDD_KI_ mice. The beneficial effect on memory was short-lived and reversible. However, the commercial peptide inhibitors are promiscuous [22] and can lead to misinterpretation of data. Thus, we treated mice with highly specific inhibitors for caspase-8 (Pen1-CrmA) or caspase-9 (Pen1-XBIR3). Only Pen1-XBIR3 reversed memory deficits. The therapeutic effect remained significant even 5 days after treatment. Therefore, we conclude that active caspase-9 plays an essential role in the pathogenesis of memory loss in FDD_KI_ mice (Figure 5A and B). Our previous findings showed that increased levels of APP metabolites derived by β-secretase processing of APP (sAPPβ and/or β-CTF) caused by loss of BRI2 protein in FDD_KI_ mice are also responsible for synaptic/memory deficits. The Danish mutation could alter APP processing and prompt caspase-9 activation *via* independent mechanisms, starting two pathogenic pathways that are necessary but not sufficient to provoke the disease (Figure 5B). Alternatively, sAPPβ and/or β-CTF may prompt caspase-9 activation, *via* a yet-to-be-defined pathway, activation of caspase-9. In turn, active caspase-9 mediates downstream events, which are still uncharacterized and may involve other caspases, leading to synaptic and memory dysfunctions (Figure 5C).

Transgenic mice overexpressing human FAD mutant *APP* (Tg2576 mice) display an Aβ-dependent enhanced caspase-3 activation, and Z-DEVD-*fmk* restores cognitive decline in Tg2576 mice [30]. It has also been shown that XBIR2, but not XBIR3, rescues the hippocampal LTP deficits induced *in vitro* by synthetic Aβ [31]. The BIR2 domain of XIAP inhibits active caspase-3 and caspase-7 [24]. Altogether, these results have lead to the conclusion that caspase-3, but not caspase-9, mediates the inhibition of LTP by synthetic Aβ. This is in contrast with the observations that only caspase-9 is hyperactive in FDD_KI_ mice and that memory deficits of FDD_KI_ mice are rescued by Z-LEHD-*fmk* and XBIR3 but not Z-DEVD-*fmk*. Those differences are consistent with the hypothesis that the deficits of FDD_KI_ mice are Aβ-independent. Based on these dissimilarities it could be argued that FDD_KI_ and Tg2576 mice represent dementias caused by distinct pathogenic mechanisms, involving either sAPPβ/β-CTF and caspase-9 or Aβ and caspase-3, respectively. Alternatively, these mice may reproduce distinct stages of a common pathway leading to human dementia. It is also possible that either FDD_KI_ or Tg2576 mice develop synaptic/memory deficits that are triggered by artificial harmful effects unrelated to the pathogenesis of human dementias. In this regard, it is important to emphasize that the mouse model that we have analyzed, unlike transgenic mice, is genetically congruous to the human disease, suggesting that the mechanisms underlying synaptic and memory impairments in FDD_KI_ mice faithfully reproduce the pathogenesis of human dementia.

When aberrant caspase-9 activation is confined to synaptic compartments, it leads to synaptic-memory deficits, as it is the case for FDD_KI_ mice (Figure 5D and E). However, if activation of caspase-9 is recurring and sustained, it may lead to dystrophy of neurites and to the demise of any given neuron in which active caspase-9 leaks into the neuronal cell body triggering effector caspases and leading to genomic DNA fragmentation (Figure 5F). Over time, these changes can result in neuronal loss and neuritic dystrophy that are typical features of advanced neurodegenerative diseases.

Our study suggests that inhibiting caspase-9 activity may be a viable therapeutic option in human dementias. Here, we have used intraventricular administration of Pen1-XBIR3 that provides direct delivery to the brain. In a previous paper we have shown that direct parenchymal or intranasal delivery of Pen1-XBIR3 is therapeutically effective in rat models of stroke [23]. From a therapeutic perspective, intranasal delivery is a very attractive treatment strategy for CNS disorders because it provides direct, noninvasive access to the brain via the olfactory pathway. Intranasal delivery combined with the cell-permeant peptide Pen1 makes Pen1-XBIR3 an attractive therapeutic compound for treatment of human dementias.

### Ethical statement regarding the use and well fare of mice

Mice were handled according to the Ethical Guidelines for Treatment of Laboratory Animals of Albert Einstein College of Medicine. The procedures were described and approved in animal protocol number 20100404.

## Materials and methods

### Mice

FDD_KI,_ mice are on a C57BL/6 J background and were generated and maintained at the Animal facility of the Albert Einstein College of Medicine. Mice were handled according to the Ethical Guidelines for Treatment of Laboratory Animals of Albert Einstein College of Medicine. The procedures were described and approved in animal protocol number 200404.

### Electrophysiology

Transverse hippocampal slices (400 μm) from 13–14 month old WT and FDD_KI_ mice were transferred to a recording chamber where they were maintained at 29°C and perfused with artificial cerebrospinal fluid (ACSF) continuously bubbled with 95% O_2_ and 5% CO_2_. The ACSF composition in mM was: 124 NaCl, 4.4 KCl, 1 Na_2_HPO_4_, 25 NaHCO_3_, 2 CaCl_2_, 2 MgSO_4_, and 10 glucose. CA1 field-excitatory-post-synaptic potentials (fEPSPs) were recorded by placing both the stimulating and the recording electrodes in CA1 stratum radiatum. After 90 minutes incubation, 10 μM Z-VAD-*fmk* was added. For LTP experiments, a 30 min baseline was recorded every minute at an intensity that evoked a response approximately 35% of the maximum evoked response. LTP was induced using a θ-burst stimulation (four pulses at 100 Hz, with bursts repeated at 5 Hz and each tetanus including one ten-burst train). Responses were recorded for 90 min after tetanization and plotted as percentage of baseline fEPSP slope. Z-VAD-*fmk* is from R&D Systems.

### Brain cannulation and injections

Dr. Xiaosong Li at the Animal Physiology core of the Albert Einstein College of Medicine surgically implanted the cannula. Using stereotaxic surgery performed under ketamine/xylazine anesthesia, mice were implanted with cannula (Plastics One Inc.) into the lateral ventricle (coordinates from bregma: A/P −0.4 mm, M/L − 1 mm, D/V −2.5 mm) or hippocampus (coordinates from bregma: A/P −2.45 mm, M/L − 1.5 mm, D/V −1.7 mm). Z-LEHD-*fmk* (800 nM), Z-DEVD*-fmk* (800 nM), or saline were delivered into the lateral ventricle at the rate of 1 μl per minute using a CMA 400 syringe pump, for a total volume of 1 μl. Pen1-XBIR3 (23 μM), Pen1-CrmA (16 μM), or saline were delivered into the lateral ventricle at a rate of 1 μl per minute using a CMA 400 syringe pump, for a total volume of 2 μl. Z-LEHD-*fmk* and Z-DEVD-*fmk* are from R&D Systems.

### *In vivo* caspase activity assay

Biotin-Val-Ala-Asp(OMe)-fluoromethylketone (bVAD; MP Biomedicals) was used as an *in vivo* molecular trap for active caspases. 5 μl of bVAD (100 nmol) was injected into one hippocampus along with a blue dye using a CMA 400 syringe pump at a rate of 1 μl per minute. Mice were sacrificed 2 hrs later, and the region with the blue dye was isolated from the rest of the hippocampus. The same region was collected on the contralateral hippocampus as the untreated/control. These regions were lysed separately in 10% glycerol, 150nM NaCl, 0.2% NP-40, 20 mM Tris–HCl (pH 7.3) with protease and phosphatase inhibitors. For bVAD-caspase complex precipitation, protein lysates were precleared by rocking with Sepharose beads (GE Healthcare) for 1 h at 4°C. Precleared lysate was centrifuged, and the supernatant was transferred to 30 μl of streptavidin-agarose beads (Sigma) and rocked gently overnight at 4°C. Beads were washed/centrifuged (300 μl washes, 5000 rpm for 1 min) 15 times with bVAD lysis buffer. After the final wash/pelleting, caspase-bVAD complexes were boiled off of streptavidin beads into 1× SDS sample buffer without reducing agent. Beads were pelleted at 14,000 rpm for 10 min, and the supernatant was transferred to a fresh tube and resolved by SDS-PAGE.

### Organotypic hippocampal slices caspase activity assay

Organotypic hippocampal slices were prepared and cultured as described previously [32]. Briefly, 400 μm slices were prepared using a tissue chopper. Slices were transferred onto a cell culture insert that was placed into a 6-well plate in an incubator with 5% CO_2_ and 78% O_2_. The plate contained 1 ml of culture media (MEM with Glutamax-1 supplemented with D-glucose, horse serum, nystatin, HEPES, EBSS, and Pen-Strep). The slices were cultured in the interface method. After 24 h of culture, the media was replaced with culture media containing 45 μM bVAD for 3 h after which the slices were collected and lysed separately in 10% glycerol, 150nM NaCl, 0.2% NP-40, 20 mM Tris–HCl (pH 7.3) with protease and phosphatase inhibitors. bVAD-caspase complex precipitation was performed by preclearing with Sepharose beads and isolation with streptavidin-agarose beads as described in the preceding chapter. After the final wash/pelleting, caspase-bVAD complexes were boiled off of streptavidin beads into 1× SDS sample buffer without reducing agent. Beads were pelleted at 14,000 rpm for 10 min, and the supernatant was transferred to a fresh tube and resolved by SDS-PAGE.

### Open field and novel object recognition

The mice were acclimated to the testing room for 30 min after being moved. Each mouse was placed into a 40 cm × 40 cm open field chamber with opaque walls, 2 ft high. Each mouse was allowed to habituate to the normal open field box for 10 min, and repeated again 24 h later, in which we manually recorded the number of entries into and time spent in the center of the locomotor arena. As previously reported [3], open field studies showed that FDD_KI_ mice have no defects in habituation, sedation, risk assessment and anxiety-like behavior in novel environments.

Novel object recognition began 24 h after the second open field session, and was performed as previously described [3,33]. Briefly, NOR consisted of two sessions 24 h apart. In the first session, the mice were placed into the open field chamber with two identical, non-toxic objects, 12 cm from the back and sidewalls of the open field box, and 16 cm apart from each other. An 8 min session, in which the time exploring each object was recorded; an area 2 cm^2^ surrounding the object is defined such that nose entries within 2 cm of the object were recorded as time exploring the object. We will refer to this as training trial. The animal was then returned to its home cage and 24 h late, placed into the open field box again. This time, there was one object identical to the previous one, and one novel object. We will refer to this as the test trial. The mice were given another 6 min to explore, and the amount of time exploring each object was recorded. Mice that spent < 7 seconds exploring the objects were omitted from the analysis [33]. Results were recorded as Time Spent Exploring each object an object discrimination ratio (ODR), which is calculated by dividing the time the mice spent exploring object 1 (for the training trial) or the novel object (for the test trial) by the total amount of time exploring the two objects.

### Synaptosome preparation and Western blot analysis

For synaptic preparations, isolated hippocampi were homogenized (w/v = 10 mg tissue/100 ml buffer) in Hepes-sucrose buffer (20 mM Hepes/NaOH pH 7.4, 1 mM EDTA, 1 mM EGTA, 0.25 M sucrose) supplemented with protease and phosphatase inhibitors. Homogenates were centrifuged at 800 g for 10 min. The supernatant (S1) was separated into supernatant (S2) and pellet (P2) by spinning at 9,200 g for 15 min. Synaptosome fractions represent: S1, post-nuclear-supernatant; S2, cytosol, soluble proteins and light membrane; P2, crude synaptosomal fraction. The S1 and P2 fractions were analyzed by western blot using the following antibodies: α-caspase-3 (9662/Cell signaling); α-caspase-6 (9762/Cell Signaling); α-caspase-8 (4790/Cell Signaling); α-caspase-9 (ab28131/Abcam. Secondary antibodies conjugated with horse-radish peroxidase are from Southern Biotechnology.

### Image scanning and analysis

Western blot images were scanned with Epson Perfection 3200 Photo scanner and were analyzed with NIH ImageJ software.

### Statistical analysis

All data are shown as mean ± s.e.m. Statistical tests included two-way ANOVA for repeated measures and t-test when appropriate. All experiments were performed in a blinded fashion.

## Competing interests

The FDD_KI_ mice are patented by the Albert Einstein College of Medicine. LD is inventor of this patent.

## Authors’ contributions

LD generated the mice. RT performed behavioral and LTP experiments. LD performed hippocampal preparations, caspases activation tests and western blot analysis. LD designed research and wrote the paper. NA and CMT provided recombinant proteins. CMT participated in designing the caspase-activation experiments. OA provided the rig for electrophysiology and helped analyzing the LTP data. All authors read and approved the final manuscript.
